# Analytical Properties of Credibilistic Expectation Functions

**DOI:** 10.1155/2014/294183

**Published:** 2014-02-26

**Authors:** Shuming Wang, Bo Wang, Junzo Watada

**Affiliations:** ^1^Department of Industrial & Systems Engineering, National University of Singapore, Block E1A No. 05-19, 1 Engineering Drive 2, Singapore 117576; ^2^Graduate School of Information, Production & Systems, Waseda University, Hibikino 2-7, Wakamatsu-ku, Kitakyshu, Fukuoka, Japan

## Abstract

The expectation function of fuzzy variable is an important and widely used criterion in fuzzy optimization, and sound properties on the expectation function may help in model analysis and solution algorithm design for the fuzzy optimization problems. The present paper deals with some analytical properties of credibilistic expectation functions of fuzzy variables that lie in three aspects. First, some continuity theorems on the continuity and semicontinuity conditions are proved for the expectation functions. Second, a differentiation formula of the expectation function is derived which tells that, under certain conditions, the derivative of the fuzzy expectation function with respect to the parameter equals the expectation of the derivative of the fuzzy function with respect to the parameter. Finally, a law of large numbers for fuzzy variable sequences is obtained leveraging on the Chebyshev Inequality of fuzzy variables. Some examples are provided to verify the results obtained.

## 1. Introduction

Possibility theory [1] aims to study the behaviors of fuzzy events via fuzzy measures. Following this pioneering work, a number of studies have been done that largely enriched the theory of possibility (see [2–6]). In the context of possibility theory, a variety of fuzzy optimization models have been developed that form a set of decision-making vehicles that are useful in tackling the situations when parameters of the decision systems carry linguistic uncertainty or vagueness (see [7]). The fuzzy optimization models with applications have already reached many areas in operations research, control, and management, such as mathematical programming (see [8–11]), regression analysis (see [12–14]), optimal control (see [15]), portfolio selection (see [16, 17]), facility location planning (see [18, 19]), and power system unit commitment (see [20]).

Expectation function of fuzzy variable is a critical and widely accepted criterion in fuzzy optimization. It is usually used to model the objective and/or constraints in the fuzzy programming with a general form of *𝔼*[*f*(*x*, *X*)], where *𝔼*[·] is the expected value operator and *f*(*x*, *X*) is function of fuzzy variable *X* and decision *x*, which could contain some profit or loss items. In the fuzzy optimization models with expectation criterion (see [10]), the analytical properties of the expectation functions play a pivotal role in model analysis and solution design. For instance, (i) the continuity conditions for *𝔼*[*f*(*x*, *X*)] of *x* make an easier way for the decision-makers to analyze the sensitivity of the objective function and/or constraint functions with respect to the decisions; (ii) the properties of differentiation could help us when designing the gradient-based algorithms for solution searching; (iii) in many process-contained fuzzy dynamic optimization problems (e.g., maximization of the long-term average lifetime of a system made of different components), the convergence properties of the sum of fuzzy variables (limit theorems or laws of large numbers) play a key role in model transformation (simplification); making use of those limit theorems, the original long-term average lifetime of all components can be expressed equivalently as the expected value of a single component which is relatively easier to compute.

To the best of our knowledge, only a limited number of studies have investigated the theoretical properties of expected values of fuzzy variables in the literature: a bounded convergence theorem was proved for expected value sequences of fuzzy variables in [21]; some properties of fuzzy expected values were discussed on the relaxations of evaluation-restrictions in [22]; some analytical formulas were derived for Max-Min operations of T-related fuzzy variables in [23]; an analytical formula was derived for the expected values of functions of continuous fuzzy variables in [24]; and a fuzzy type Wald's Equation was derived for the expected value of the sum of a fuzzy number of fuzzy variables in [25].

The present paper is devoted to deriving several new analytical properties of fuzzy (credibilistic) expectation functions, along the above-mentioned three directions, that is, the continuity, the differentiation, and limit theorems. The paper is organized in the following manner.  Section 2 recalls some preliminaries on a fuzzy expected value operator and several necessary results. In Section 3, some continuity theorems on the continuity, upper semicontinuity, and lower semicontinuity conditions for the expectation functions are derived. Furthermore, a differentiation formula of expectation functions is derived in Section 4.  Section 5 proves a law of large numbers for fuzzy variable sequences. Finally, a brief summary is covered in Section 6.

## 2. Preliminaries

Given a universe Γ, an *ample field* (see [26]) *𝒜* on Γ is a class of subsets of Γ that contains Γ and is closed under arbitrary unions and complementation in Γ. Let Pos be a set function defined on the ample field *𝒜*. The set function Pos is said to be a *possibility measure* if it satisfies the following conditions:

(P1) Pos(*∅*) = 0, and Pos(Γ) = 1;
(P2) Pos (⋃_*i*∈*ℐ*_ *A*_*i*_) = sup⁡_*i*∈*ℐ*_Pos(*A*_*i*_) for any subclass {*A*_*i*_ | *i* ∈ *ℐ*} of *𝒜*, where *ℐ* is an arbitrary index set.

Triplet (Γ, *𝒜*, Pos) is called a *possibility space*, which also was named a *pattern space* by Nahmias [4].

Leveraging on possibility measure, a self-dual set function Cr, called *credibility measure* [10], is defined on the possibility space (Γ, *𝒜*, Pos) as

(1) $$\begin{array}{c} \text{Cr}\left(A\right):=\frac{1+\text{Pos}\left(A\right)-\text{Pos}\left(A^{c}\right)}{2}, \end{array}$$

for any *A* ∈ *𝒜*, where *A* ^*c*^ is the complement of *A*. A function *X* : Γ → *ℜ* is said to be a fuzzy variable defined on Γ, if {*γ* ∈ Γ | *X*(*γ*) ≤ *t*} ∈ *𝒜* for every *t* ∈ *ℜ*. The possibility distribution of *X*, denoted by *μ*_*X*_, is defined by *μ*_*X*_(*x*) = Pos{*γ* ∈ Γ | *X*(*γ*) = *x*}. Moreover, the credibility distribution of *X* is defined as

(2) $$\begin{array}{c} G_{X}\left(x\right):=\text{Cr}\left\{\gamma \in \Gamma \;|\;X\left(\gamma \right)\geq x\right\},\;x\in ℜ. \end{array}$$

For more detailed discussions on credibility distribution of fuzzy variable, one may refer to [27–29].

Let *X* be a fuzzy variable with possibility distribution *μ*_*X*_(*x*), the support of *X* is defined by

(3) $$\begin{array}{c} \Xi :=\mathrm{cl}\left\{x\in ℜ\;|\;\mu_{X}\left(x\right)>0\right\}, \end{array}$$

where cl⁡*A* is the closure of set *A* ⊂ *ℜ*. Obviously, if we denote *Ξ*_*γ*_ : = {*γ* ∈ Γ | *X*(*γ*) ∈ *Ξ*}, then Cr(*Ξ*_*γ*_) = 1.

**Definition 1 (see [10])**

Let *X* be a fuzzy variable defined on a possibility space (Γ, *𝒜*, Pos). The expected value of *X* is defined as

(4) $$\begin{array}{c} 𝔼\left[X\right]=\int_{0}^{\infty }\text{Cr}\left\{X\geq r\right\}\text{d}r-\int_{-\infty }^{0}\text{Cr}\left\{X\leq r\right\}\text{d}r, \end{array}$$

provided that one of the two integrals is finite, where Cr is the credibility measure given by (1).

Moreover, the expected value of *X* is said to be finite provided

(5) $$\begin{array}{c} \max\left\{\int_{0}^{\infty }\text{Cr}\left\{X\geq r\right\}\text{d}r,\int_{-\infty }^{0}\text{Cr}\left\{X\leq r\right\}\text{d}r\right\}<\infty . \end{array}$$

In this case, fuzzy variable *X* is said to be integrable.

**Definition 2 (see [10])**

Let *X* be a fuzzy variable with finite expected value *e*. The variance of *X* is defined by *𝕍*[*X*] = *𝔼*[(*X* − *e*)^2^].

Furthermore, fuzzy variables *X*_1_, *X*_2_,…, *X*_*n*_ are said to be min-related if and only if

(6) $$\begin{array}{c} \text{Pos}\left\{X_{i}\in B_{i},\;i=1,2,\ldots ,n\right\}=\underset{1\leq i\leq n}{\min}\;\text{Pos}\left\{X_{i}\in B_{i}\right\} \end{array}$$

for any sets *B*_1_, *B*_2_ …, *B*_*n*_ of *ℜ*. For any min-related fuzzy variables *X* and *Y* with finite expected values, it has been proved that the following linear additivity holds in expected value (see [10]):

(7) $$\begin{array}{c} 𝔼\left[X+Y\right]=𝔼\left[X\right]+𝔼\left[Y\right]. \end{array}$$

For a sequence of fuzzy variables, we have the following convergence modes.

**Definition 3 (see [30])**

Suppose that {*X*_*n*_} is a sequence of fuzzy variables defined on the possibility space (Γ, *𝒜*, Pos). We say that the sequence {*X*_*n*_} converges in credibility to *X* if, for any *ϵ* > 0,

(8) $$\begin{array}{c} \underset{n\to \infty }{\lim}\text{Cr}\left\{\gamma \in \Gamma \;|\;\left|X_{n}\left(\gamma \right)-X\left(\gamma \right)\right|\geq \epsilon \right\}=0, \end{array}$$

and is denoted as $X_{n}\overset{\text{Cr}}{\to }X$.

**Definition 4 (see [30])**

Let (Γ, *𝒜*, Pos) be a possibility space on which fuzzy variables {*X*_*n*_} and *X* are defined. If, for every *ϵ* > 0, there exits *B* ∈ *𝒜*, such that Cr(*B*) < *ϵ* and

(9) $$\begin{array}{c} \underset{n\to \infty }{\lim}\underset{\;\gamma \in \Gamma \setminus B}{\sup}\left|X_{n}\left(\gamma \right)-X\left(\gamma \right)\right|=0. \end{array}$$

Then we say that sequence {*X*_*n*_} converges almost uniformly to *X*, and is denoted as $X_{n}\overset{\text{a}.\text{u}.}{\to }X$.

**Definition 5 (see [27])**

Let {*X*_*n*_} and *X* be fuzzy variables whose credibility distributions are {*G*_*X*_*n*__} and *G*_*X*_, respectively. We say that sequence {*X*_*n*_} converges in distribution to *X*, denoted by $X_{n}\overset{\text{d}.}{\to }X$ if {*G*_*X*_*n*__} converges to *G*_*X*_ on the set of continuity points of *G*_*X*_.

**Theorem 6 (see [30])**

Suppose that {*X*_*n*_} is a fuzzy variable sequence defined on possibility space (Γ, *𝒜*, Pos). Sequence {*X*_*n*_} that converges almost uniformly to *X* implies that {*X*_*n*_} converges in distribution to *X*.

A sequence {*X*_*k*_} of fuzzy variables is said to be uniformly essentially bounded (see [21]) if there is a positive number *a* such that *G*_*X*_*k*__(−*a*) = 1, and *G*_*X*_*k*__(*a*) = 0 for *k* = 1,2,…. For the convergence of the expected value sequences, we have the following bounded convergence theorem.

**Theorem 7 (see [21])**

Suppose {*X*_*n*_} is a sequence of uniformly essentially bounded fuzzy variables. If $X_{n}\overset{\text{Cr}}{\to }X$, then one has

(10) $$\begin{array}{c} \underset{n\to \infty }{\lim}𝔼\left[X_{n}\right]=𝔼\left[X\right]. \end{array}$$

## 3. Continuity

This section is intended to discuss the continuity of credibilistic expectation function *𝔼*[*f*(*t*, *X*)], where parameter *t* ∈ *ℜ*, and *f*(*t*, *x*) are a real-valued bivariate function on *ℜ* ^2^. First of all, for a family of fuzzy variables {*X*_*t*_, *t* ∈ *𝒯*}, where *𝒯* is any index set, we have the following lemma.

**Lemma 8**

Let {*X*_*t*_, *t* ∈ *𝒯*} be a family of fuzzy variables. Assume that there are integrable fuzzy variables *η* and *ζ* such that

(11) $$\begin{array}{c} G_{\eta }\leq G_{X_{t}}\leq G_{\zeta } \end{array}$$

except on an at most countable set (or e.c., for short) for any *t* ∈ *𝒯*. If $X_{t}\overset{d.}{\to }X_{t_{0}}(t\to t_{0})$, then

(12) $$\begin{array}{c} \underset{t\to t_{0}}{\lim}𝔼\left[X_{t}\right]=𝔼\left[X_{t_{0}}\right]. \end{array}$$

**Proof**

Since $X_{t}\overset{\text{d}.}{\to }X_{t_{0}}\;\;(t\to t_{0})$, that is, lim⁡_*t*→*t*_0__ *G*_*X*_*t*__(*r*) = *G*_*X*_*t*_0___(*r*), where *r* is any continuity point of *G*_*X*_*t*_0___, therefore, for any sequence {*t*_*n*_}, *t*_*n*_ → *t*_0_  (*n* → *∞*), we have

(13) $$\begin{array}{c} \underset{n\to \infty }{\lim}G_{X_{t_{n}}}\left(r\right)=G_{X_{t_{0}}}\left(r\right), \end{array}$$

where *r* is any continuity point of *G*_*X*_*t*_0___. It follows from Lebesgue's dominated convergence theorem that

(14) $$\begin{array}{c} \underset{n\to \infty }{\lim}𝔼\left[X_{t_{n}}\right]=𝔼\left[X_{t_{0}}\right], \end{array}$$

and the arbitrary of {*t*_*n*_} proves the lemma.

**Theorem 9**

Let *X* be a fuzzy variable with support *Ξ*, and *f*(*t*, *x*) a uniformly continuous real-valued function on [*a*, *b*] × *Ξ* ⊂ *ℜ* ^2^. If there exist integrable fuzzy variables *η* and *ζ* such that

(15) $$\begin{array}{c} G_{\eta }\leq G_{f(t,X)}\leq G_{\zeta }\;e.c.,\;\;t\in \left[a,b\right], \end{array}$$

then *𝔼*[*f*(*t*, *X*)] is continuous on [*a*, *b*].

**Proof**

For every *t* ∈ [*a*, *b*], by the uniform continuity of *f*(*t*, *x*) on [*a*, *b*] × *Ξ* we have that, for every *ϵ* > 0, there corresponds a *δ* > 0 such that

(16) $$\begin{array}{c} \left|f\left(s,X\left(\gamma \right)\right)-f\left(t,X\left(\gamma \right)\right)\right|<\epsilon ,\;\forall \gamma \in \Xi_{\gamma }, \end{array}$$

provided |*s* − *t*| < *δ*. Noting that Cr(*Ξ*_*γ*_) = 1, we have

(17) $$\begin{array}{c} \underset{s\to t\;}{\lim}\underset{\;\gamma \in \Xi_{\gamma }}{\sup}\left|f\left(s,X\left(\gamma \right)\right)-f\left(t,X\left(\gamma \right)\right)\right|=0. \end{array}$$

That is,

(18) $$\begin{array}{c} f\left(s,X\right)\overset{\text{a}.\text{u}.}{⟶}f\left(t,X\right)\;\left(s⟶t\right) \end{array}$$

which implies

(19) $$\begin{array}{c} f\left(s,X\right)\overset{\text{d}.}{⟶}f\left(t,X\right)\;\left(s⟶t\right). \end{array}$$

By the assumptions of the theorem, Lemma 8 deduces that

(20) $$\begin{array}{c} \underset{s\to t}{\lim}𝔼\left[f\left(s,X\right)\right]=𝔼\left[f\left(t,X\right)\right]. \end{array}$$

The proof of the theorem is complete.

We note that any bounded and closed set on *ℜ* ^2^ is compact; therefore, the following corollary is valid naturally.

**Corollary 10**

Suppose that *X* is a fuzzy variable with bounded support *Ξ*, and real-valued function *f*(*t*, *x*) is continuous on [*a*, *b*] × *Ξ* ⊂ *ℜ* ^2^. If there exist integrable fuzzy variables *η* and *ζ* such that

(21) $$\begin{array}{c} G_{\eta }\leq G_{f(t,X)}\leq G_{\zeta }\;e.c.,\;\;t\in \left[a,b\right], \end{array}$$

then *𝔼*[*f*(*t*, *X*)] is continuous on [*a*, b].

**Theorem 11**

Let *X* be a fuzzy variable with support *Ξ*. If real-valued function *f*(*t*, *x*) is uniformly continuous and bounded on [*a*, *b*] × *Ξ* ⊂ *ℜ* ^2^, then *𝔼*[*f*(*t*, *X*)] is continuous on [*a*, *b*].

**Proof**

On the one hand, *f*(*t*, *x*) is bounded [*a*, *b*] × *Ξ*, which implies that there is a positive number *M* such that |*f*(*t*, *X*(*γ*))| ≤ *M*, for all *t* ∈ [*a*, *b*] and *γ* ∈ *Ξ*_*γ*_. Hence, the family of fuzzy variables {*f*(*t*, *X*), *t* ∈ [*a*, *b*]} is uniformly essentially bounded.

On the other hand, from the proof of Theorem 9, it follows from the uniformly continuity of *f*(*t*, *x*) on [*a*, *b*] × *Ξ* that

(22) $$\begin{array}{c} f\left(s,X\right)\overset{\text{a}.\text{u}.}{⟶}f\left(t,X\right)\;\left(s⟶t\right), \end{array}$$

which implies

(23) $$\begin{array}{c} \underset{s\to t}{\lim}\text{Cr}\left\{\left|f\left(s,X\right)-f\left(t,X\right)\right|\geq \epsilon \right\}=0. \end{array}$$

Combing the above two aspects, Theorem 7 implies

(24) $$\begin{array}{c} \underset{s\to t}{\lim}𝔼\left[f\left(s,X\right)\right]=𝔼\left[f\left(t,X\right)\right], \end{array}$$

which proves the theorem.

**Theorem 12**

Let *X* be a fuzzy variable with support *Ξ* and *t*_0_ ∈ [*a*, *b*] ⊂ *ℜ*. Assume that *f*(*t*_0_, *x*) satisfies for every *ϵ* > 0, there is *δ* > 0 such that

(25) $$\begin{array}{c} f\left(s,x\right)\leq f\left(t_{0},x\right)+\epsilon ,\;\forall x\in \Xi , \end{array}$$

provided |*s* − *t*_0_| < *δ*. Then *𝔼*[*f*(*t*, *X*)] is upper semicontinuous at *t*_0_.

**Proof**

Since for every *ϵ* > 0, there is a *δ* > 0 such that |*s* − *t*_0_| < *δ*, one has

(26) $$\begin{array}{c} f\left(s,x\right)\leq f\left(t_{0},x\right)+\epsilon ,\;\forall x\in \Xi , \end{array}$$

which implies

(27) $$\begin{array}{c} f\left(s,X\left(\gamma \right)\right)\leq f\left(t_{0},X\left(\gamma \right)\right)+\epsilon ,\;\forall \gamma \in \Xi_{\gamma }. \end{array}$$

Recalling that Cr(*Ξ*_*γ*_) = 1, we have Cr(Γ∖*Ξ*_*γ*_) = 0. Therefore, for every *r* > 0, we obtain

(28) Cr{f(s,X)≥r}=Cr(Ξγ∩{f(s,X)≥r})≤Cr(Ξγ∩{f(t0,X)+ϵ≥r})=Cr{f(t0,X)+ϵ≥r}.

Similarly, we could get

(29) $$\begin{array}{c} \text{Cr}\left\{f\left(s,X\right)\leq r\right\}\geq \text{Cr}\left\{f\left(t_{0},X\right)+\epsilon \leq r\right\} \end{array}$$

for any *r* < 0.

Thus, combining (28) and (29), we have

(30) 𝔼[f(s,X)]=∫0∞Cr{f(s,X)≥r}dr−∫−∞0Cr{f(s,X)≤r}dr≤∫0∞Cr{f(t0,X)+ϵ≥r}dr −∫−∞0Cr{f(t0,X)+ϵ≤r}dr=𝔼[f(t0,X)+ϵ]=𝔼[f(t0,X)]+ϵ,

which implies the upper semicontinuity of *𝔼*[*f*(*t*, *X*)] at *t*_0_. The proof of the theorem is complete.

**Theorem 13**

Let *X* be a fuzzy variable with support *Ξ* and *t*_0_ ∈ [*a*, *b*]. Assume that *f*(*t*_0_, *x*) satisfies for every *ϵ* > 0, there is a *δ* > 0 such that

(31) $$\begin{array}{c} f\left(s,x\right)\geq f\left(t_{0},x\right)-\epsilon ,\;\forall x\in \Xi , \end{array}$$

provided |*s* − *t*_0_| < *δ*. Then *𝔼*[*f*(*t*, *X*)] is lower semicontinuous at *t*_0_.

**Proof**

The theorem can be proved by the same logic as that used in Theorem 12.

## 4. Differentiation

In this section, for a function of fuzzy variable *f*(*t*, *X*_*t*_) with parameter *t*, we will discuss under which conditions the differentiation formula

(32) $$\begin{array}{c} \nabla_{t}𝔼\left[f\left(t,X_{t}\right)\right]=𝔼\left[\nabla_{t}f\left(t,X_{t}\right)\right] \end{array}$$

is true, where

(33) $$\begin{array}{c} \nabla_{t}f\left(t,x\right):=\frac{\partial f\left(t,x\right)}{\partial t}. \end{array}$$

**Theorem 14**

Letting *X*_*t*_ be a fuzzy variable with support *Ξ* for any real number *t* ∈ [*a*, *b*], fuzzy variables *X*_*t*_ and *X*_*s*_ are mutually min-related and identically distributed for any different *t*, *s* ∈ [*a*, *b*]. Function *f*(*t*, *x*):[*a*, *b*] × *Ξ* → *ℜ* is differentiable in *t* ∈ [*a*, *b*] for any *x* ∈ *Ξ*, and *f*(*t*, *X*_*t*_) is an integrable fuzzy variable for any *t* ∈ [*a*, *b*]. if the following two conditions hold:

(i) for every *ϵ* > 0 it corresponds a *δ* > 0 such that
  (34) $$\begin{array}{c} \left|\nabla_{s}f\left(s,x\right)-\nabla_{t}f\left(t,x\right)\right|<\epsilon ,\;\forall x\in \Xi , \end{array}$$
  provided 0 < |*s* − *t*| < *δ*;
(ii) there exist integrable fuzzy variables *η* and *ζ* such that
  (35) $$\begin{array}{c} \eta \left(\gamma \right)\leq \nabla_{s}f\left(s,X_{s}\right)\left(\gamma \right)\leq \zeta \left(\gamma \right),\;\forall \gamma \in \Xi_{\gamma }, \end{array}$$
  provided *s* ∈ [*a*, *b*].

Then ∇_*t*_ *𝔼*[*f*(*t*, *X*_*t*_)] exists and

(36) $$\begin{array}{c} \nabla_{t}𝔼\left[f\left(t,X_{t}\right)\right]=E\left[\nabla_{t}f\left(t,X_{t}\right)\right]. \end{array}$$

**Proof**

Under condition (i), we first claim the following result:

(37) $$\begin{array}{c} \underset{s\to t}{\lim}\frac{f\left(s,x\right)-f\left(t,x\right)}{s-t}=\nabla_{t}f\left(t,x\right), \end{array}$$

uniformly on *Ξ*.

Consider the difference quotients

(38) $$\begin{array}{c} \psi \left(s,x\right):=\frac{f\left(s,x\right)-f\left(t,x\right)}{s-t} \end{array}$$

for 0 < |*s* − *t*| < *δ*. Since *f*(*t*, *x*) is differentiable on [*a*, *b*], by Lagrange mean value theorem, there corresponds to each (*s*, *x*) a number *u* between *t* and *s* such that

(39) $$\begin{array}{c} \psi \left(s,x\right)=\frac{f\left(s,x\right)-f\left(t,x\right)}{s-t}=\nabla_{u}f\left(u,x\right). \end{array}$$

Hence, condition (i) implies that

(40) $$\begin{array}{c} \left|\psi \left(s,x\right)-\nabla_{t}f\left(t,x\right)\right|<\epsilon ,\;\forall x\in \Xi , \end{array}$$

as |*s* − *t*| < *δ*. That is,

(41) $$\begin{array}{c} \underset{s\to t}{\lim}\psi \left(s,x\right)=\nabla_{t}f\left(t,x\right) \end{array}$$

uniformly on *Ξ*.

Next, we prove

(42) $$\begin{array}{c} \psi \left(s,X\right)\overset{\text{d}.}{⟶}\nabla_{t}f\left(t,X\right)\;\left(s⟶t\right). \end{array}$$

By (41), we know

(43) lim⁡s→tψ(s,Xs(γ))=lim⁡s→tf(s,Xs(γ))−f(t,Xt(γ))s−t=∇tf(t,Xt(γ))

for all *γ* ∈ *Ξ*_*γ*_. That is, *ψ*(*s*, *X*_*s*_) converges to ∇_*t*_ *f*(*t*, *X*_*t*_) uniformly on *Ξ*_*γ*_, as *s* → *t*.

Since Cr(*Ξ*_*γ*_) = 1, we get

(44) $$\begin{array}{c} \psi \left(s,X\right)\overset{\text{a}.\text{u}.}{⟶}\nabla_{t}f\left(t,X_{t}\right)\;\left(s⟶t\right), \end{array}$$

which by Theorem 6 implies

(45) $$\begin{array}{c} \psi \left(s,X\right)\overset{\text{d}.}{⟶}\nabla_{t}f\left(t,X_{t}\right)\;\left(s⟶t\right). \end{array}$$

Applying Lagrange mean value theorem again, for each pair (*γ*, *s*), there corresponds a *u* ∈ [*a*, *b*] such that

(46) $$\begin{array}{c} \psi \left(s,X_{s}\left(\gamma \right)\right)=\nabla_{u}f\left(u,X_{u}\left(\gamma \right)\right). \end{array}$$

Since

(47) $$\begin{array}{c} \eta \leq \nabla_{u}f\left(u,X_{u}\left(\gamma \right)\right)\leq \zeta ,\;\forall \gamma \in \Xi_{\gamma }, \end{array}$$

provided *u* ∈ [*a*, *b*], we get

(48) $$\begin{array}{c} \eta \leq \psi \left(s,X_{s}\left(\gamma \right)\right)\leq \zeta ,\;\forall \gamma \in \Xi_{\gamma }, \end{array}$$

which implies that

(49) $$\begin{array}{c} G_{\eta }\leq G_{\psi (s,X_{s})}\leq G_{\zeta }. \end{array}$$

Noting that *f*(*t*, *X*_*t*_) and *f*(*s*, *X*_*t*_) are integrable and min-related fuzzy variables, it has

(50) $$\begin{array}{c} 𝔼\left[\psi \left(s,X_{s}\right)\right]=\frac{𝔼\left[f\left(s,X_{s}\right)\right]-𝔼\left[f\left(t,X_{t}\right)\right]}{s-t}. \end{array}$$

It follows from condition (ii) and Lemma 8 that

(51) ∇t𝔼[f(t,Xt)]=lim⁡s→t𝔼[f(s,Xs)]−𝔼[f(t,Xt)]s−t=lim⁡s→t𝔼[ψ(s,Xs)]=𝔼[∇tf(t,Xt)].

The desired result follows and the proof of the theorem is completed.

The following simple example verifies the result of Theorem 14.

**Example 15**

Given a positive triangular fuzzy variable *Y* = (*a*_1_, *a*_2_, *a*_3_), *a*_1_ > 0, we define a family of mutually min-related fuzzy variables *X*_*t*_, *t* ∈ [*a*, *b*], *a* > 0 with the same possibility distribution of *Y*. The support of *X*_*t*_ is *Ξ* = [*a*_1_, *a*_3_], which is a bounded and closed set of *ℜ*. Define the function *f*(*t*, *x*) = *t* ^2^ · (*x* − *t*). Let us verify the result of Theorem 14.

Apparently, *f*(*t*, *x*) = *t* ^2^ · (*x* − *t*) is differentiable on [*a*, *b*] with respect to *t*. Also note that

(52) 𝔼[f(t,Xt)]=E[t2·((a1,a2,a3)−t)]=t2((a1+2a2+a3)4−t),

and *f*(*t*, *X*_*t*_) is an integrable fuzzy variable for each *t* ∈ [*a*, *b*].

As for conditions (i) and (ii), on the one hand, since

(53) $$\begin{array}{c} \left[a,b\right]\times \Xi =\left[a,b\right]\times \left[a_{1},a_{3}\right] \end{array}$$

is a compact set of *ℜ* ^2^and *f*(*t*, *x*) = *t* ^2^ · (*x* − *t*) is a uniformly continuous function on [*a*, *b*] × *Ξ*, condition (i) holds.

On the other hand, we note that, for any *t* ∈ [*a*, *b*],

(54) $$\begin{array}{c} \nabla_{t}f\left(t,X_{t}\right)=\frac{\partial f\left(t,X_{t}\right)}{\partial t}=2tX_{t}-3t^{2}; \end{array}$$

we then have

(55) $$\begin{array}{c} 2aa_{1}-3b^{2}\leq \nabla_{t}f\left(t,X_{t}\right)\leq 2ba_{3}-3a^{2},\;\forall \gamma \in \Xi_{\gamma }; \end{array}$$

then condition (ii) also holds.

Finally, by (52) and (54), we can obtain

(56) ∇t𝔼[f(t,Xt)]dt=t·(a1+2a2+a3)2−3t2=𝔼[∇tf(t,Xt)],

which verifies the result of Theorem 14.

From the above example, it is natural to have the following corollary.

**Corollary 16**

Let *X*_*t*_ be a fuzzy variable with bounded support *Ξ* for any *t* ∈ [*a*, *b*], and fuzzy variables *X*_*t*_ and *X*_*s*_ are mutually min-related and identically distributed for any different *t*, *s* ∈ [*a*, *b*]. Function *f*(*t*, *x*):[*a*, *b*] × *Ξ* → *ℜ* is differentiable in *t* ∈ [*a*, *b*] for any *x* ∈ *Ξ*, and *f*(*t*, *X*_*t*_) is an integrable fuzzy variable for each *t* ∈ [*a*, *b*]. Under the following two conditions:

(i) ∇_*t*_ *f*(*t*, *x*) is continuous on [*a*, *b*] × *Ξ*,
(ii) there exist integrable fuzzy variables *η* and *ζ* such that
  (57) $$\begin{array}{c} \eta \leq \nabla_{s}f\left(s,X_{s}\right)\leq \zeta ,\;\forall \gamma \in \Xi_{\gamma }, \end{array}$$
  provided *s* ∈ [*a*, *b*]; one has that ∇_*t*_ *𝔼*[*f*(*t*, *X*_*t*_)] exists and
  (58) $$\begin{array}{c} \nabla_{t}𝔼\left[f\left(t,X_{t}\right)\right]=E\left[\nabla_{t}f\left(t,X_{t}\right)\right]. \end{array}$$

**Proof**

Since *Ξ* is bounded and [*a*, *b*] × *Ξ* is a compact set in *ℜ* ^2^, condition (i) implies the uniformly continuity of ∇_*t*_ *f*(*t*, *x*) on [*a*, *b*] × *Ξ*. Therefore, for every *ϵ* > 0 there is a *δ* > 0 such that

(59) $$\begin{array}{c} \left|\nabla_{s}f\left(s,x\right)-\nabla_{t}f\left(t,x\right)\right|<\epsilon \end{array}$$

for all *x* ∈ *Ξ* and *s* ∈ (*t* − *δ*, *t* + *δ*). Thus, applying Theorem 14 proves the corollary.

## 5. A Law of Large Numbers

This section focuses on the law of large numbers for fuzzy variable sequences. Analogously to the case of random variables, there is also a parallel important inequality—Chebyshev Inequality—for fuzzy variables.

**Theorem 17 (see [27], Chebyshev Inequality)**

Let *X* be a fuzzy variable whose variance *𝕍*[*X*] exists. Then for any given number *t* > 0, one has

(60) $$\begin{array}{c} \text{ Cr}\left\{\left|X-𝔼\left[X\right]\right|\geq t\right\}\leq \frac{𝕍\left[X\right]}{t^{2}}. \end{array}$$

**Theorem 18**

Let {*X*_*n*_} be a sequence of fuzzy variables. If *𝕍*[*X*_1_ + ⋯+*X*_*n*_] < *∞* for every *n*, and

(61) $$\begin{array}{c} \underset{n\to \infty }{\lim}\frac{1}{n^{2}}𝕍\left[\sum_{k=1}^{n}X_{k}\right]=0, \end{array}$$

then one has

(62) $$\begin{array}{c} \frac{1}{n}\sum_{k=1}^{n}X_{k}-\frac{1}{n}𝔼\left[\sum_{k=1}^{n}X_{k}\right]\overset{\text{ Cr }}{⟶}0. \end{array}$$

**Proof**

Since

(63) $$\begin{array}{c} 𝕍\left[\frac{1}{n}\sum_{k=1}^{n}X_{k}\right]=\frac{1}{n^{2}}𝕍\left[\sum_{k=1}^{n}X_{k}\right], \end{array}$$

by Chebyshev Inequality (60) and condition (61), for any *ϵ* > 0, we have

(64) Cr{|1n∑k=1nXk−1n𝔼[∑k=1nXk]|≥ϵ} ≤1ϵ2𝕍[1n∑k=1nXk] =1ϵ2n2𝕍[∑k=1nXk]⟶0 (n⟶∞).

The required result follows.

**Example 19**

For a sequence of (min-related) triangular fuzzy variables

(65) $$\begin{array}{c} X_{k}=\left(1-\frac{1}{k^{2}},1,1+\frac{1}{k^{2}}\right) \end{array}$$

for *k* = 1,2,…, let us verify the result of Theorem 18.

To begin, we verify the condition that *𝕍*[*X*_1_ + ⋯+*X*_*n*_] < *∞* for every *n*. Since, for each *n*,

(66) $$\begin{array}{c} 𝔼\left[X_{1}+\cdots +X_{n}\right]=𝔼\left[\sum_{k=1}^{n}\left(1-\frac{1}{k^{2}},1,1+\frac{1}{k^{2}}\right)\right]=n, \end{array}$$

we have

(67) $$\begin{array}{c} 𝕍\left[X_{1}+\cdots +X_{n}\right]=𝔼\left[{\left(\sum_{k=1}^{n}X_{k}-n\right)}^{2}\right]=𝔼\left[\Sigma^{2}\right], \end{array}$$

where

(68) $$\begin{array}{c} \Sigma =\left(-\sum_{k=1}^{n}\frac{1}{k^{2}},0,\sum_{k=1}^{n}\frac{1}{k^{2}}\right). \end{array}$$

Denoting *K*(*n*): = ∑_*k*=1_^*n*^1/*k* ^2^, we have

(69) $$\begin{array}{c} \Sigma =\left(-K\left(n\right),0,K\left(n\right)\right); \end{array}$$

then the possibility distribution of Σ can be written as

(70) $$\begin{array}{c} \text{Pos}\left\{\Sigma =x\right\}=\{\begin{array}{cc} \frac{\left(K\left(n\right)-\left|x\right|\right)}{K\left(n\right)}, & x\in \left(-K\left(n\right),K\left(n\right)\right)\\ 0, & \text{otherwise}. \end{array} \end{array}$$

Furthermore, the possibility distribution of Σ^2^ can be expressed as

(71) $$\begin{array}{c} \text{Pos}\left\{\Sigma^{2}=x\right\}=\{\begin{array}{cc} \frac{\left(K\left(n\right)-\sqrt{x}\right)}{K\left(n\right)}, & x\in \left(0,K{\left(n\right)}^{2}\right)\\ 0, & \text{otherwise}, \end{array}\\ \text{Cr}\left\{\Sigma^{2}\geq x\right\}=\{\begin{array}{cc} \frac{\left(K\left(n\right)-\sqrt{x}\right)}{2K\left(n\right)}, & x\in \left(0,K{\left(n\right)}^{2}\right)\\ 0, & \text{otherwise}. \end{array} \end{array}$$

Noting that

(72) $$\begin{array}{c} K\left(n\right)=\sum_{k=1}^{n}\frac{1}{k^{2}}↑\frac{\pi^{2}}{6},\;\left(n⟶\infty \right), \end{array}$$

for any *n*, we have

(73) 𝕍[X1+⋯+Xn]=𝔼[Σ2]=∫0K(n)2K(n)−x2K(n)dx=16K(n)2=∑k=1n16k2<π236.

On the other hand, we have

(74) $$\begin{array}{c} \underset{n\to \infty }{\lim}\frac{1}{n^{2}}𝕍\left[\sum_{k=1}^{n}X_{k}\right]=\underset{n\to \infty }{\lim}\frac{1}{n^{2}}\sum_{k=1}^{n}\frac{1}{6k^{2}}\leq \underset{n\to \infty }{\lim}\frac{\pi^{2}}{6n^{2}}=0. \end{array}$$

Combining (73) and (74), the conditions of Theorem 18 hold.

Now, for any *ϵ* > 0, we calculate

(75) $$\begin{array}{c} \text{Cr}\left\{\left|\frac{1}{n}\sum_{k=1}^{n}X_{k}-\frac{1}{n}𝔼\left[\sum_{k=1}^{n}X_{k}\right]\right|\geq \epsilon \right\}, \end{array}$$

which is equal to

(76) $$\begin{array}{c} \text{Cr}\left\{\left|\left(-\frac{K\left(n\right)}{n},0,\frac{K\left(n\right)}{n}\right)\right|\geq \epsilon \right\}. \end{array}$$

Define

(77) $$\begin{array}{c} ℰ\left(n\right):=\left(-\frac{K\left(n\right)}{n},0,\frac{K\left(n\right)}{n}\right); \end{array}$$

note that *K*(*n*)/*n* → 0  (*n* → *∞*); we have

(78) lim⁡n→∞Cr{|ℰ(n)|≥ϵ}≤lim⁡n→∞Pos{|ℰ(n)|≥ϵ}=lim⁡n→∞max⁡{1−ϵK(n)/n,0}=0.

That is,

(79) $$\begin{array}{c} \frac{1}{n}\sum_{k=1}^{n}X_{k}-\frac{1}{n}𝔼\left[\sum_{k=1}^{n}X_{k}\right]\overset{\text{Cr}}{⟶}0. \end{array}$$

The result of Theorem 18 is verified.

Clearly, we have the following corollary for min-related and identically distributed fuzzy variable sequences.

**Corollary 20**

Let {*X*_*n*_} be a sequence of min-related and identically distributed fuzzy variables. If  *𝕍*[*X*_1_ + ⋯+*X*_*n*_] < *∞* for every *n*, and

(80) $$\begin{array}{c} \underset{n\to \infty }{\lim}\frac{1}{n^{2}}𝕍\left[\sum_{k=1}^{n}X_{k}\right]=0, \end{array}$$

then one has

(81) $$\begin{array}{c} \frac{1}{n}\sum_{k=1}^{n}X_{k}\overset{\text{ Cr }}{⟶}𝔼\left[X_{1}\right]. \end{array}$$

**Proof**

For min-related and identically distributed fuzzy variables *X*_1_, *X*_2_,…, we have

(82) $$\begin{array}{c} 𝔼\left[\frac{1}{n}\sum_{k=1}^{n}X_{k}\right]=\frac{1}{n}\sum_{k=1}^{n}𝔼\left[X_{k}\right]=𝔼\left[X_{1}\right], \end{array}$$

where *n* ≥ 2. This proves the corollary.

## 6. Concluding Remarks

In the present study, we investigated some analytical properties of the credibilistic expectation functions of fuzzy variables. The major new results obtained can be summarized as follows.

(i) Several continuity theorems including the conditions of upper semicontinuity, lower semicontinuity, and continuity (Theorems 9–13) were established for expectation function *𝔼*[*f*(*t*, *X*)].
(ii) The differentiability of expected value *𝔼*[*f*(*t*, *X*_*t*_)] was discussed and a differentiation formula
  (83) $$\begin{array}{c} \nabla_{t}𝔼\left[f\left(t,X_{t}\right)\right]=𝔼\left[\nabla_{t}f\left(t,X_{t}\right)\right] \end{array}$$
  was derived (Theorem 14).
(iii) A law of large numbers was proved for fuzzy variable sequences (Theorem 18).

There is much room for further development based on the present study. First, this paper only discussed the properties of expectation functions in single variable case, which could be extended to a multivariate case for broader potential applications. Furthermore, some other analytical properties, such as integration, could also be interesting issues for future studies. Finally, the results obtained in the present work within the scope of fuzzy variable are worth being considered with a generalization to the case of hybrid uncertainty with fuzzy random variables.
